# Sorafenib and Treg Differentiation: A Paradigm Shift in Hepatocellular Carcinoma Management

**DOI:** 10.1016/j.jcmgh.2025.101478

**Published:** 2025-02-22

**Authors:** Hanyang Liu

**Affiliations:** Thoracic and GI Malignancies Branch, Center for Cancer Research, National Cancer Institute, National Institutes of Health, Bethesda, Maryland

Hepatocellular carcinoma (HCC) is among the most challenging cancers to manage, given its late-stage diagnosis and limited treatment options.^1^, ^2^ Sorafenib, a multikinase inhibitor, has been a mainstay therapy for advanced HCC.^3^^,^^4^ Despite its initial promise, sorafenib’s efficacy is frequently undermined by resistance, leading to poor outcomes for patients.^5^ The development of resistance to sorafenib has been attributed to tumor-intrinsic factors and tumor microenvironment (TME) alterations.^6^ Previous studies have highlighted the role of immune cells, such as myeloid-derived suppressor cells and tumor-associated macrophages, in mediating resistance.^7^^,^^8^ Shen et al^8^ reveal that sorafenib induces Treg differentiation via VEGFR/AKT/Foxo1 signaling, creating an immunosuppressive TME and reducing its efficacy. By dampening anti-HCC immunity, Tregs pose a critical barrier to treatment success, highlighting the need for therapies targeting cancer cells and the immune landscape.

This study demonstrates that sorafenib modulates the VEGFR/AKT/Foxo1 signaling axis, leading to increased differentiation of Treg cells. Notably, the inhibition of this pathway reversed Treg induction and enhanced anti-HCC immunity, highlighting a promising therapeutic target. Furthermore, sorafenib-induced Treg cells exhibit enhanced immunosuppressive activity, reducing the proliferation and cytotoxicity of CD8^+^ T cells. This suggests that the expansion of Tregs significantly contributes to the immune evasion observed in sorafenib-treated HCC. In addition, the combination of sorafenib with an anti-CD25 antibody or the Foxo1 inhibitor AS1842856 not only mitigated Treg-mediated suppression but also improved therapeutic efficacy in preclinical models. These findings provide a strong rationale for clinical trials exploring similar combinatorial strategies.

Constructively, this study provides insights into anti-HCC immunity in sorafenib therapy. However, there are certain limitations to be improved. The study's reliance on preclinical models, predominantly murine models and in vitro experiments, may limit its ability to fully replicate the complexity of human HCC and its TME. Furthermore, there is an absence of the long-term VEGFR/AKT/Foxo1 inhibition assessment on systemic immune function. Prolonged immune modulation could have unintended consequences, such as autoimmune responses or disruption of immune homeostasis. Although Treg cells are extensively studied in HCC,^9^^,^^10^ less attention was paid to other immune cell types impacted by sorafenib (eg, dendritic cells and B lymphocytes). A more holistic understanding of the immune landscape could provide additional insights. However, this study did not include comprehensive clinical trials to confirm the efficacy of the proposed combination therapies in a real-world setting. Last, the broad inhibition of VEGFR and downstream pathways by sorafenib and the studied inhibitors may have effects beyond Treg modulation, which are not fully characterized in this work.

Turning the limitation into inspiration, further ponderations and efforts are needed to address the previously mentioned research gaps. To substantiate the findings' relevance, extensive validation in diverse cohorts of patients with HCC is imperative. The combination of sorafenib with Treg-targeting agents, such as anti-CD25 antibodies or Foxo1 inhibitors, should be prioritized in clinical trials. Subsequent studies should examine how sorafenib affects other immune cells, such as macrophages, neutrophils, and natural killer cells, which also contribute to the TME. Besides, cancer-stroma-immune cell interactions imprint the intricate HCC TME and uncertain responses to therapies.^11^^,^^12^ A more in-depth investigation into the systemic effects and off-target impacts of sorafenib and VEGFR/AKT/Foxo1 pathway inhibitors is essential to ensure their safety and efficacy. Considering the crucial influences of senescence occurrence on tuing cancer cell fates and immunogencity, the senescence inducing or clearance therpies are emerging, providing promising combinative strategies in HCC treatment.^13^, ^14^, ^15^ Further research should focus on optimizing dosage, timing, and combination strategies to balance efficacy with potential toxicities in treatments that include immune modulators alongside sorafenib. Studies that track the durability of the immune response and overall survival following combination therapies would provide crucial insights into their long-term viability.

Taken together, this study revealed a novel regulatory mechanism that sorafenib facilitates immunosuppressive TME and therapeutic resistance in HCC. Restraining Tregs or the Foxo1 activity enhances antitumor immunity and therapeutic outcomes of sorafenib in preclinical models. Tregs suppress CD8^+^ T cell activity, emphasizing the need to counteract this immune evasion. The findings underscore the potential of combining sorafenib with Treg-targeting agents, identifying VEGFR/AKT/Foxo1 as a pivotal pathway for therapeutic intervention and biomarker development to enhance HCC patient outcomes by overcoming drug resistance.
